# Stroke in a child with pulmonary tuberculosis and pleural effusion—An important clue for the diagnosis of disseminated central nervous system tuberculosis

**DOI:** 10.1002/ccr3.6945

**Published:** 2023-02-10

**Authors:** Nagendra Chaudhary, Binod Kumar Gupta, Astha Poudel, Manish Kafle, Neeva Singh, Hanshmani Prasad Chaudhary

**Affiliations:** ^1^ Department of Pediatrics Universal College of Medical Sciences Bhairahawa Nepal

**Keywords:** CNS tuberculosis, pleural effusion, pulmonary tuberculosis, stroke in tuberculosis

## Abstract

Central nervous system tuberculosis (CNS‐TB) is one of the most devastating and life‐threatening conditions having high mortality and morbidity. Here, we report a 12‐year‐old child with pulmonary tuberculosis and pleural effusion presenting with ischemic stroke as an important manifestation of central nervous system tuberculosis.

## Abstract 2

Rapid and accurate diagnoses with timely treatment are the best ways to improve the survival rate of CNS‐TB. This report focuses the need to consider CNS‐TB in the differential diagnosis of children with stoke living in areas of high tuberculosis.

## 1 INTRODUCTION

Tuberculosis (TB) is an important public health concern especially in low‐ and middle‐income countries (LMICs). Among all other types of TB, central nervous system tuberculosis (CNS‐TB) is one of the most devastating and life‐threatening condition having high mortality and morbidity even with or without treatment.^1^, ^2^ The frequency of CNS‐TB is 1%–2% of all active TB and 6%–10% of extra‐pulmonary TB.^3^ CNS‐TB comprises three clinical manifestations, that is, tubercular meningitis, intracranial tuberculoma, and spinal tubercular arachnoiditis. Therefore, the key to prevent poor outcomes due to CNS‐TB is early and prompt diagnosis and its appropriate treatment.

Stroke is highly prevalent in developed countries and is one of the second most leading cause of death worldwide. The prevalence in developing countries is also rising which has varied etiologies.^4^

In developing nations, apart from many other causes of stroke, CNS‐TB is also one of the important etiological factors of stroke. A recent report from Sudan by Kheir et al has reported CNS‐TB as an unusual causes of stroke in a 12‐year‐ old boy with Down syndrome.^5^

The objective of this article is to report a case of ischemic stroke as an important manifestation of cerebral tuberculosis in a child who had pulmonary tuberculosis with effusion. Early and prompt identification of CNS‐TB in such cases can guide the clinician to consider longer duration of anti‐tubercular drugs (9–12 months rather than 6 months in pulmonary TB) which will help to decrease the mortality and morbidity due to CNS‐TB.

## 2 CASE HISTORY AND EXAMINATION

A 12‐year‐old male child presented to the pediatric casualty with complaints of fever and productive cough for 7 days followed by sudden onset of inability to move his left upper and lower limbs for 1 day. He also had facial deviation towards the right side. There was no history of difficulty in breathing or hemoptysis. He did not have any abnormal body movement, loss of consciousness, altered sensorium, projectile vomiting, or slurring of speech. No any history of urinary incontinence, trauma, skin rashes, or jaundice was noticed. Family history of tuberculosis or any contact was not present.

On examination, there was no pallor, icterus, cyanosis, clubbing, or lymphadenopathy. His weight was 25 kg, height was 130 cm, and body mass index was 14.8 kg/m^2^. Respiratory system examination revealed respiratory rate of 22 breaths per minute, decreased vocal tactile fremitus below right 3rd intercostal spaces, dull note on percussion at 4th intercostal space and reduced breath sounds over right mammary, infra‐mammary, axillary, and infra‐axillary spaces. There were also crepitations present over the left infra‐axillary and infra‐scapular regions. Central nervous system examination revealed Glasgow coma scale of 15/15 and the child was oriented to time, place, and person. Left‐sided lower motor neuron type of 7th nerve palsy was noted (obliteration of left nasolabial folds, deviation of angle of mouth to the right side of face, drooling of saliva from the left side of mouth, and inability to close the left eye tightly). Motor system examination showed normal power (5/5) in right upper and lower limbs while absent power in left upper and lower limbs (0/5). Deep tendon reflexes on the left side were exaggerated whereas bilateral plantars were flexors. Sensory system examination was normal, and there was no any signs of meningeal irritation.

## 3 INVESTIGATIONS

**FIGURE 1:**
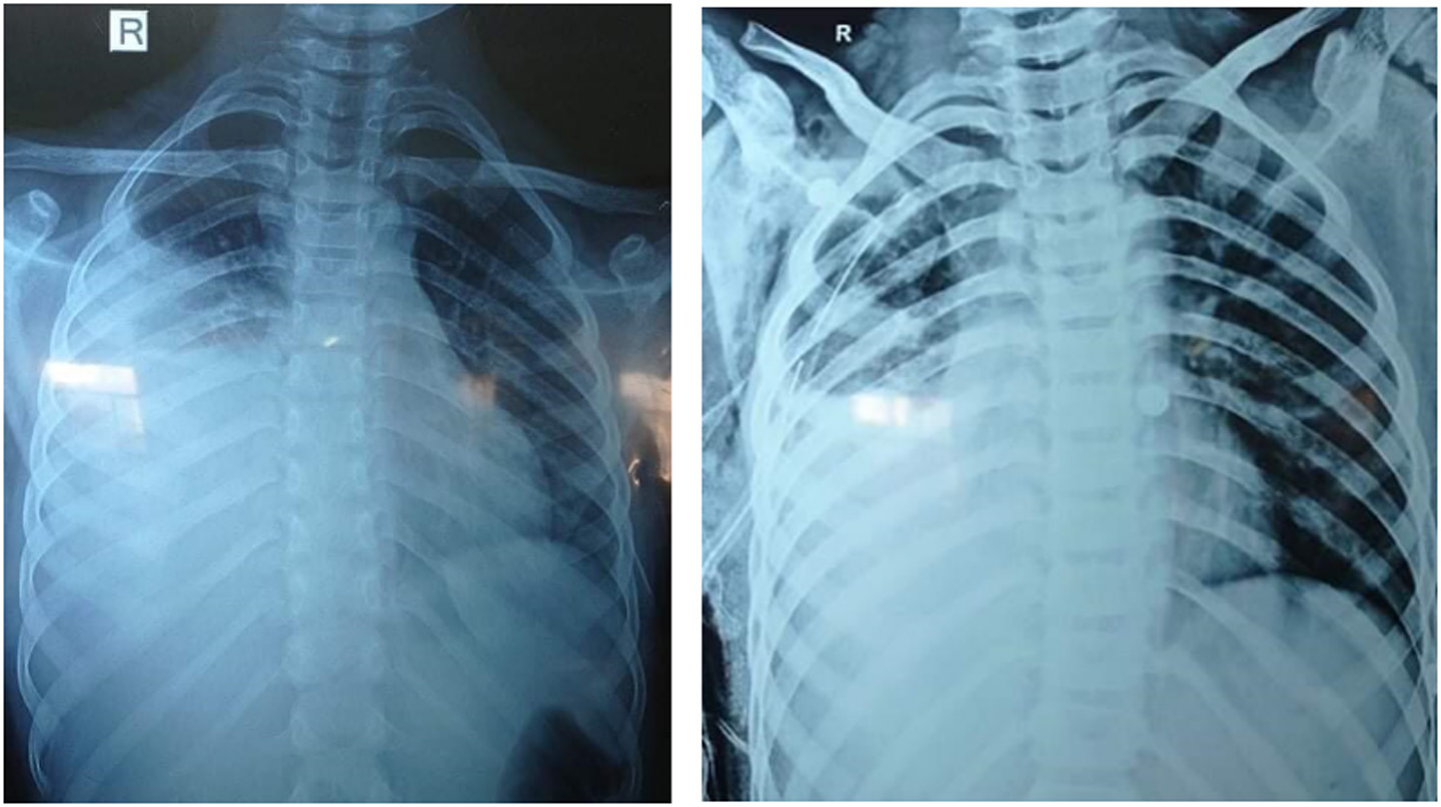
(A) Chest X‐ray showing right sided pleural effusion; (B) X‐ray showing pleural effusion with chest tube insertion.

Routine investigations were planned and sent. Complete blood count showed hemoglobin‐11.6 g/dL, total leukocyte count‐ 9600 cells/mm^3^, platelet count‐ 530,000 cells/mm^3^, and differential counts (N_72_, L_10_, E_8_, and M_10_). ESR was 55 mm/1st hour. Total serum protein and LDH were 5.5 g/dL and 1638 U/L, respectively. Renal function test and serum electrolytes were in the normal range. Chest X‐ray showed right‐sided pleural effusion (Figure 1A,B).

Pleural fluid analysis showed clear yellowish fluid with absent cobweb and predominance of lymphocytes (94%) with normal gram staining. Pleural fluid sugar and protein were 75 mg/dL and 4.1 g/dL, respectively, with no growth on culture. Pleural fluid LDH was raised (1318 U/L), and ADA was also raised (34.2 U/L). Cerebrospinal fluid analysis showed cell count, glucose, and protein in normal limits. Pleural fluid and CSF for Gene X‐pert MTB/RIF was negative. Mantoux test was negative. SARS‐CoV‐2 (2019‐nCoV) antigen test was negative. Mycobacterium tuberculosis was detected in two samples of three daily morning sputum samples sent for Ziehl Neelsen (ZN) staining.

*Work up for stroke—*Prothrombin time (PT) and ANA was normal. Echocardiography was done to rule out heart disease which was normal. Doppler of bilateral carotid arteries was also normal. Ultrasonography of abdomen and pelvis was normal. Scrub typhus (IgG and IgM) serology was negative.

**FIGURE 2:**
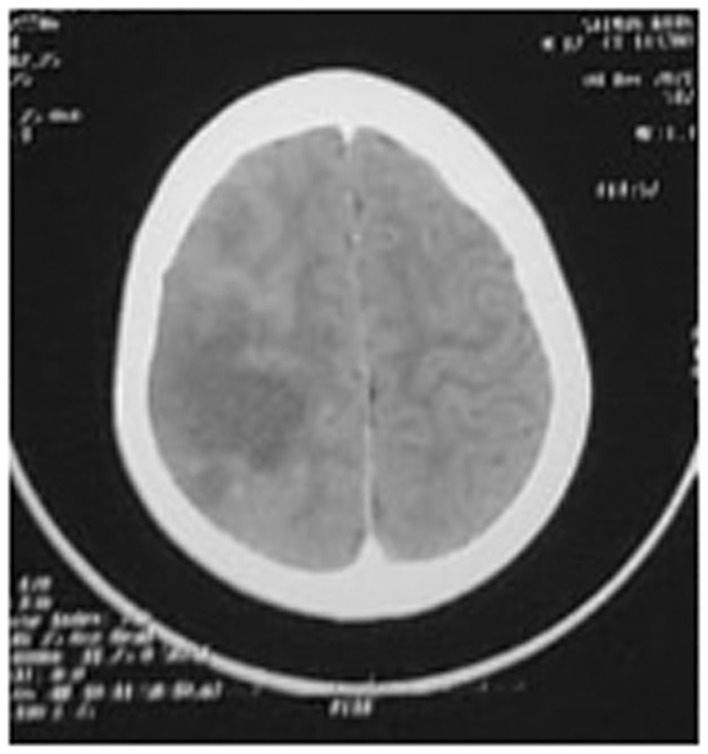
CT head showing hypodense area on right temporo‐parietal region.

As the child had left‐sided hemiplegia, neuroimaging was planned. Neuroimaging (CT scan head) showed non‐enhancing geographical ill‐defined hypo dense area in the cortical and subcortical region of right fronto‐parietal lobe with mass effect in the form of effacement to adjacent sulcal and gyral space and loss of gray and white matter differentiation (Figure 2). The diagnosis of pulmonary tubercular effusion with stroke was considered based on these investigations.

**FIGURE 3:**
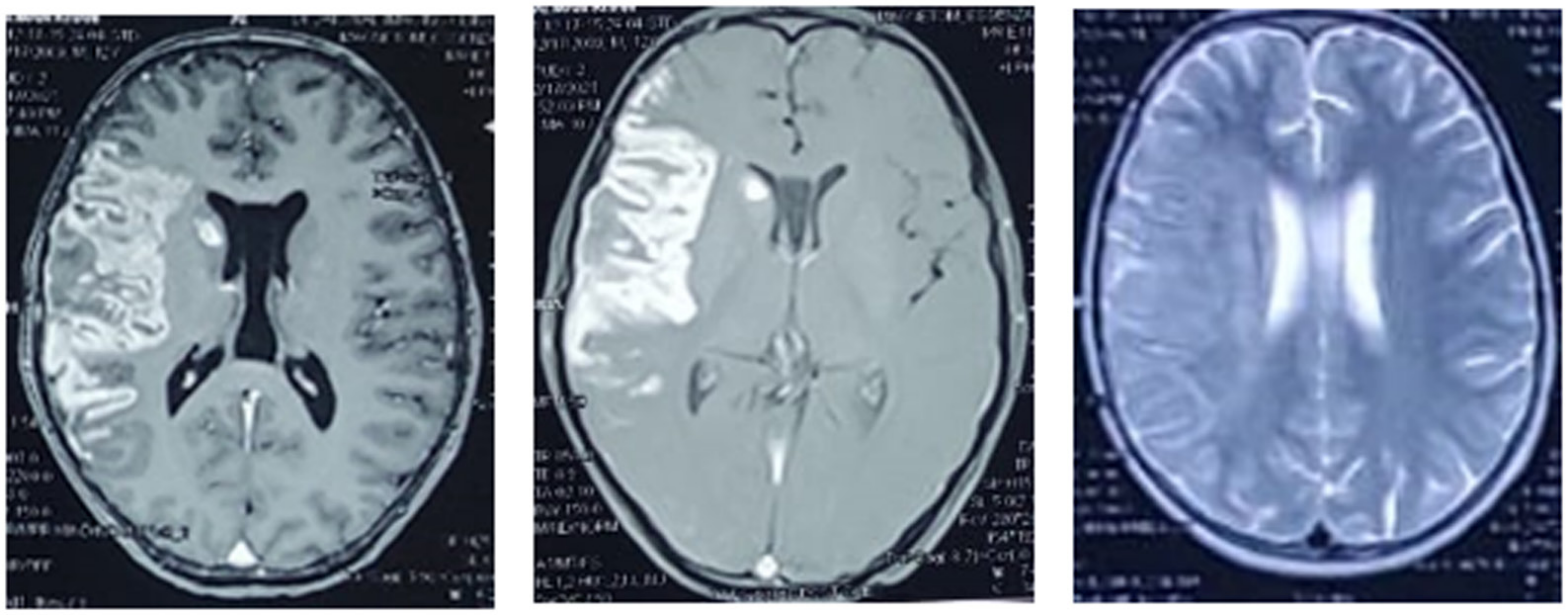
(A–C). MRI Brain showing restriction diffusion at right lentiform nucleus, caudate nucleus, centrum semiovale and corona radiata which appeared hyper‐intense on T2/FLAIR and hypo‐intense on T1 with patchy contrast enhancement in centrum semiovale suggestive of vasculitic infarct. Few T2 hypo‐intense homogeneous enhancing lesions seen in region of caudate nucleus suggestive of granulomatous lesion. Large area of T2/FLAIR hyperintensity in cortical, subcortical and deep white matter of right fronto‐parietal region with heterogeneous contrast enhancement involving the meninges, cortical and subcortical regions with area of FLAIR sulcal hyperintensity suggestive of meningoencephalitis.

Further, MRI brain was also considered (Figure 3) which showed a large area of restriction diffusion at right lentiform nucleus, caudate nucleus, centrum semiovale, and corona radiata which appeared hyper‐intense on T2/FLAIR and hypo‐intense on T1 with patchy contrast enhancement in centrum semiovale suggestive of vasculitic infarct. Few T2 hypo‐intense homogeneous enhancing lesions were also noted scattered in region of caudate nucleus suggestive of granulomatous lesion (tuberculoma). Also, large area of T2/FLAIR hyperintensity was noted in cortical, subcortical, and deep white matter of right fronto‐parietal region with heterogeneous contrast enhancement involving the meninges, cortical, and subcortical regions with area of FLAIR sulcal hyperintensity suggestive of meningoencephalitis (secondary of Koch).

Finally, the child was diagnosed as disseminated tuberculosis (pulmonary tuberculosis with pleural effusion + CNS tuberculosis) and was started on anti‐tubercular drugs (2HRZE + 10HRE).

## 4 TREATMENT, OUTCOME, AND FOLLOW‐UP

The child was admitted to the pediatric intensive care unit and started on broad spectrum intravenous antibiotics (piperacliin‐tazobactum and vancomycin) for massive pleural effusion of possible bacterial etiology. Around 300 mL of pleural fluid tapping was done under aseptic precautions but there was no improvement noticed on repeat chest X‐ray done the day after. Therefore, underwater sealed chest tube drainage (Figure 1B) was considered which was removed when three consecutive pleural fluid drain volume was <50 mL (on 6th day of chest tube insertion). The child was also started on aspirin after neurological consultation in view of ischemic cerebrovascular accident. Once the diagnosis of pulmonary TB and CNS‐TB was confirmed, the child was started on anti‐tubercular drug regimen along with steroids (1 mg/kg/day of oral prednisolone for 6 weeks followed by tapering its dose over next 2 weeks) as per the national tuberculosis control program guidelines.

Broad spectrum intravenous antibiotics were given for 3 weeks followed by 1 week of oral antibiotics. Subsequent, chest X‐ray at discharge was done which was normal. The child was discharged after 3 weeks of hospital admission. The child was followed up subsequently at 1 month and 3 months. The child did not encounter any side effects of anti‐tubercular drugs. The child at 3‐month follow‐up was able to walk although he had hemiplegic gait which was improving subsequently. Follow‐up MRI brain could not be done due to the patient's financial constraints.

## 5 DISCUSSION

Central nervous system involvement in disseminated tuberculosis is not a common manifestation and requires a high index of suspicion to diagnose it. The most important symptoms in children with CNS‐TB are fever, headache, vomiting, lassitude, night sweats, and anorexia. Once the disease progresses, they can even have altered sensorium, and urinary retention.^6^

The severity of illness in CNS‐TB has been assessed using the Glasgow Coma Scale by medical research council which consists of three stages.^7^ The child does not have any local nerve damage or change in consciousness during stage 1 but can have altered sensorium and nerve damage (hemiplegia and cranial nerve paralysis) at stage 2. At 3rd stage, the child has stupor, coma, seizures, paralysis, hemiplegia, or paraplegia. The important signs of CNS‐TB are presence of meningism (Kernig's sign, Brudzinski's sign, and intracranial hypertension) which was not present in our case. Therefore, it is difficult to diagnose CNS‐TB in such children with stroke solely depending on clinical presentations and signs, because other CNS infections may also have similar symptoms. On the contrary, thromboembolic phenomenon due to cardiac etiology, arterio‐venous malformations, and autoimmune disorders also contributes as important etiological factors for pediatric stroke. Therefore, a series of more precise detection method is urgently needed to assist the diagnosis.

Cerebrospinal fluid analysis is considered as an important diagnostic tool for the diagnosis of TB meningitis. A mononuclear pleocytosis, low glucose and chloride levels and high protein concentration are supportive of TB meningitis.^8^, ^9^ ADA is increased in CNS infectious diseases, but its rise is more remarkable in TBM. In the present case, although the cell count, glucose level, and protein concentration were normal, ADA levels were high which prompt us to think of other investigative tools to rule out CNS‐TB in this child with stroke and pulmonary TB.

CT scan and MRI are being commonly used imaging methods for CNS‐TB.^10^ Neuroimaging findings highly suggestive of CNS‐TB are basal meningeal enhancement, hydrocephalus, tuberculoma, or infarction.^11^ The specificity of CT scan is relatively low for the diagnosis of CNS‐TB whereas MRI can provide more detailed diagnostic information.^12^ In the present case also, we could not see any other findings suggestive of CNS‐TB in CT scan except for infarct. In such cases, one should be cautious to draw final conclusion because other infectious or non‐infectious diseases may also display similar characteristic images. CSF analysis and detection of *M. tuberculosis* by various methods can help to diagnose TB meningitis. MRI with diffusion weighting is superior in detecting infarction in such situations where diagnostic dilemma exists.^13^ In our case, we were able to diagnose CNS‐TB in a child with pulmonary TB with pleural effusion after MRI scan although CT scan and CSF analysis were inconclusive.

Routine neuroimaging for all children with pulmonary TB is not recommended. For example, if the child in the present case did not have stroke as a neurological finding, one would not have performed CT/MRI scan and have easily missed CNS‐TB. Therefore, neurological finding in a child with pulmonary TB provides important clue for the diagnosis of CNS‐TB. Nicolls et al has reported symptomatic intracranial tuberculoma during the therapy for pulmonary tuberculosis when the child developed neurological symptoms like agitation, and inability to speak.^14^

Medical therapy is currently the recommended treatment for disseminated tuberculosis. The regimen of isoniazid, rifampicin, ethambutol, and pyrazinamide along with steroids usually results in clinical cure of CNS‐TB.^15^ The child in our case also responded well to the medical treatment, gained his lower limb power, and was able to walk at 1 month follow‐up.

## 6 CONCLUSION

The present case emphasizes the need to consider CNS‐TB in the differential diagnosis of children with stoke living in areas of high tuberculosis incidence. One should look for other diagnostic modalities for TB even if some of them do not support CNS‐TB. Rapid and accurate diagnosis and timely treatment are the best ways to improve the survival rate of CNS‐TB.

## AUTHOR CONTRIBUTIONS

**Nagendra Chaudhary:** Supervision; writing – original draft; writing – review and editing. **Binod Kumar Gupta:** Supervision; writing – original draft; writing – review and editing. **Astha Poudel:** Writing – review and editing. **Manish Kafle:** Writing – review and editing. **Neeva Singh:** Writing – review and editing. **Hanshmani Prasad Chaudhary:** Writing – review and editing.

## FUNDING INFORMATION

This research did not receive any specific grant from funding agencies in the public, commercial, or not‐for‐profit sectors.

## CONFLICT OF INTEREST STATEMENT

None.

## CONSENT

Written informed consent was obtained from father of the patient to publish this report in accordance with the journal's patient consent policy.

## Data Availability

The data that support the findings of this study are available from the corresponding author upon reasonable request.
